# Humor creation during efforts to find humorous cognitive reappraisals of threatening situations

**DOI:** 10.1007/s12144-019-00296-9

**Published:** 2019-05-24

**Authors:** Ilona Papousek, Christian Rominger, Elisabeth M. Weiss, Corinna M. Perchtold, Andreas Fink, Kurt Feyaerts

**Affiliations:** 1 Institute of Psychology, University of Graz, Graz, Austria; 2 Department of Linguistics, University of Leuven, Leuven, Belgium

**Keywords:** Humor creation, Cognitive reappraisal, Fluency, Funniness, Corpus analysis, Linguistic mechanisms

## Abstract

This interdisciplinary study examined the structure of humor creation in the specific context of efforts to positively reappraise stressful situations for effective coping. In a sample of *n* = 101 participants, a performance test was used to assess the quantity (fluency, number of generated ideas that qualified as humor) and quality (rated funniness) of humor creation in cognitive reappraisal. Linguistic mechanisms were identified and quantified using cognitive-linguistic methods of corpus analysis, and their employment was correlated with humor production performance on the level of the individual. Almost all individuals were able to come up with reappraisal ideas that qualified as humorous. Depressive symptoms, a negative mood state, and high perceptions of threat did not compromise the participants’ capability to create humor. Individuals who were more serious-minded as a trait produced ideas that were rated as less funny, but their basic ability to create humor was unaffected. Metonymy (a contiguity-based principle of meaning extension) emerged as by far the most prominent semantic mechanism in the creation of humorous re-interpretations. Furthermore, its use was related to good humor creation performance in terms of quantity and quality, which is in line with its assumed importance in the extension of meaning in general and the creation of humor in particular. Further effective linguistic mechanisms and conceptual phenomena were identified. The empirical data may be valuable for the development of interventions involving the creation of humorous ideas for cognitive reappraisal.

## Introduction

Humor creation denotes the ability to spontaneously create humor (Ruch and Heintz 2019). Despite widespread beliefs that humor creation entails several benefits to the individual, empirical research has primarily focused on the comprehension and appreciation of humor, while relatively little research has been done on humor creation (see Ruch and Heintz 2019 for overview). The present study addressed humor creation in an affective context. Specifically, we examined humor production during cognitive reappraisal of threatening situations in terms of quantity and quality of the humor, and studied the use and usefulness of specific linguistic mechanisms in the creation of humor. Additionally, it was investigated whether depressive symptoms, high levels of seriousness, a bad mood, or high perception of threat may hinder humor creation in the context of cognitive reappraisal.

Cognitive reappraisal refers to deliberately viewing an emotionally evocative event from a different perspective and re-interpreting its meaning, thereby changing its emotional impact (Lazarus and Alfert 1964; Lazarus and Folkman 1984). Based on the widely accepted view that an individual’s appraisal of a stressful situation in terms of meaning and significance, rather than the situation itself, determines the emotional response (Lazarus 1993; see also Ellsworth and Scherer 2003), cognitive reappraisal is regarded a powerful coping strategy. Meta-analyses of relevant empirical research confirmed the particular effectiveness of cognitive reappraisal in dealing with stressful situations (Augustine and Hemenover 2009; Webb et al. 2012). The creation of humor has long been ascribed a valuable role in the cognitive (re-)appraisal of threatening situations (Abel 2002; Kuiper and Martin 1998; Kuiper et al. 1995). More recent empirical studies confirmed the use of humor as an effective means in deliberate cognitive reappraisal efforts (Kugler and Kuhbandner 2015; Samson and Gross 2012; Samson et al. 2014). The effectiveness of humor in the context of cognitive reappraisal was attributed to the perspective change on a stressful situation. This perspective change facilitates greater emotional distance and exchanges negative for positive emotions due to absurd elements and incongruity-resolution, finally resulting in less threatening but still realistic appraisals of the situation (Abel 2002; Geisler and Weber 2010; Kuiper et al. 1995; Samson and Gross 2012; Samson et al. 2014).

In previous empirical investigations directly addressing the use of humor in cognitive reappraisal, negative pictures were provided with pre-specified humorous interpretations (Kugler and Kuhbandner 2015), or participants were instructed to humorously reappraise negative pictures in their minds (Samson and Gross 2012; Samson et al. 2014). The impact of these manipulations was assessed by participants’ ratings of their feelings. From these studies, it seems possible to mix cognitive reappraisal with humor, and that the incorporation of humor in cognitive reappraisal can have a positive impact. The actual feasibility of humor creation in the context of cognitive reappraisal and its structure have not been analyzed to date.

In the present study, a performance test was used to assess humor creation in cognitive reappraisal as an ability in the narrower sense (referring to what individuals can do at their best; Cronbach 1970). To this end, the Reappraisal Inventiveness Test, a standardized performance test for cognitive reappraisal generation (Weber et al. 2014; see also Fink et al. 2017; Papousek et al. 2017; Perchtold et al. 2018; Rominger et al. 2018) was modified to use it with a humor instruction. Participants were confronted with self-relevant threatening situations and were instructed to create as many *humorous* cognitive reinterpretations as possible that may downregulate their experienced stress and anxiety (cf. Perchtold et al. 2019). The vignettes of the Reappraisal Inventiveness Test were carefully designed and evaluated to meet the following criteria (de Assuncao et al. 2015; Weber et al. 2014): (1) In line with cognitive emotion theories (Ortony et al. 1988), the vignettes address basic anxieties in the general population (e.g., darkness) and elicit mild to moderate anxiety. The latter is crucial because, if emotional intensity is too high, cognitive reappraisal is unlikely to work (Sheppes et al. 2014). (2) The depicted situations offer realistic subjective possibilities to downregulate the experienced anxiety. (3) The situations were designed to elicit anxiety specifically, and not other potentially related emotions like anger or frustration. For the purposes of the present study, this operationalization gives the items of the Reappraisal Inventiveness Test several advantages over other material such as autobiographical recall of actually experienced situations. While arguably more self-relevant to the respective individual, autobiographically retrieved situations are not comparable as regards emotional intensity, context, controllability/manageability, and specificity of elicited emotions. Finally, to enable meaningful comparisons of reappraisal and humor production performance and usage of linguistic structures between individuals (in this study) and study populations (in future studies), the use of standardized test items is imperative.

In detail, research aims and hypotheses were as follows. Firstly, the feasibility of creating humor in the context of cognitive reappraisal efforts in threatening situations was examined on a descriptive level. To this end, participants’ written responses to the test items were analyzed in terms of the quantity and quality of the produced humor. Secondly, it was tested if humor creation in this particular affective context may be compromised by traits and states of the individual for which such effects may be expected. Core depressive symptoms, also in subclinical manifestations, include impaired experience of pleasure and related impaired generation of positive affect. This entails reduced interest in stimuli associated with otherwise positive or rewarding experiences, which may also affect humor production (Dunn 2012; Falkenberg et al. 2011b), presumably primarily the quantity of humor creation. Depressed persons are also less able to use positive imagery in a way that improves their mood (Grol et al. 2017). As positive imagery is vital in the generation of humorous positive re-interpretations of stressful circumstances, this deficit may impair the individual’s fluency in humor creation (quantity), but also, perhaps in particular, its quality. Similar relations may apply to a momentary negative mood state. Previous research showed a low but statistically significant negative correlation of dispositional bad mood with quality of humor in a cartoon-caption task (Ruch and Köhler 2007). Taken together, we expected lower quantity and quality of humor production in individuals scoring higher on depressive symptoms as well as in persons who were in a more negative mood state.

More serious-minded individuals are characterized by a sober, non-playful attitude (Ruch and Köhler 2007; Ruch et al. 1996) and, as such, may be less likely to use irony or create remote relations that look like nonsense in the first place. In a cartoon-caption task, seriousness was negatively correlated with the quality and quantity of punch line production (Ruch and Köhler 2007). However, relevant attitudes may affect particularly the quality of responses. People with more positive attitudes toward creativity are more likely to make remote associations, that is, connections between seemingly unrelated things (Acar and Runco 2014). Ideas covering a greater associative distance are perceived as more original, or more funny in the case of humorous ideas (e.g., Tschacher et al. 2015). More serious-minded individuals, who are less playful and value humor less, may also accept remote associations to a lesser degree and possibly invest less resources to generate more remote (funnier) associates (Acar and Runco 2014). Thus, we expected individuals scoring high on seriousness primarily to produce humor lower in quality, and the quantity of humor to be less affected. Finally, for similar reasons as were suggested in the context of cognitive reappraisal in general, one may argue that humorous re-interpretations may be more difficult in situations that are experienced as very stressful. In the case of high subjective perceptions of threat, the stronger focus on the anxiety-eliciting features of the situation may impede the ability to switch focus and, as a result, the ability to come up with humorous re-interpretations of that situation (Sheppes et al. 2014; Weber et al. 2014). However, as the test items were purposely designed to elicit mild to moderate anxiety only, we expected only small effects as regards correlations between the rated intensity of threat and humor creation.

Thirdly, in the current study for the first time cognitive reappraisal ideas classified as humor were linguistically examined by identifying and quantifying a number of potential linguistic mechanisms for effective humor creation, to estimate their prominence in the spontaneous production of humor in this context. To that end, the present study adopted a socio-cognitive account of meaning, which no longer considers meaning to be an independent system-inherent value, but as a dynamic process of conceptualization, largely determined by contextual and situational aspects of the usage-event (e.g., Barlow and Kemmer 2000; Langacker 2001). Of particular interest to our purpose, then, was the identification of the semantic and conceptual relationships,^1^ which turned out to be most relevant for the process of meaning extension in our study: the interpretational shift from the initial scene as a threat to its subsequent interpretation as a potentially humorous scene.

Since this process of reinterpretation as it shapes the cognitive reappraisal ideas classified as humor is by no means fundamentally different from any other process of meaning extension, we hypothesized that most of the cases of humorous reappraisal would involve at least one of the common mechanisms of semantic construal (Croft and Cruse 2004): metaphor, metonymy, and polysemy. More specifically, based on previous linguistic research findings regarding the principles of spontaneous humor creation (Feyaerts 2013), we expected that especially metonymy would manifest itself as the most prominent semantic mechanism underlying the generation of humorous cognitive reappraisals (Feyaerts and Brône 2005; Tabacaru and Feyaerts 2016).

Metaphor can be characterized as the semantic relationship which, on the basis of a conceptualized similarity, holds between different meanings in different domains of experience. Typical examples, illustrating the classic view on metaphor as a rhetorical element, are expressions as in (1), which are commonly characterized as comparisons without any comparative element (‘like’), as a consequence of which one entity is being mapped directly onto another. Other examples like the ones in (2) illustrate the more recent cognitive approach to metaphor as a conceptual phenomenon, which also identifies metaphors in large and fundamental areas of human understanding of the world. Accordingly, the expressions in (2) demonstrate our conceptualization of quantity in terms of verticality, whereas the examples in (3) express human conceptualization of time in terms of horizontality as yet another spatial dimension.

1. Achilles is a lion / you are a cow! / my life is a horrible mess
2. Boeing shares are going down after the crash in Ethiopia / prices are going up again / the employment rate is still climbing
3. Winter is approaching fast / Let’s leave these problems behind / Let’s move on with this! / She has a bright future ahead of her

Metonymy qualifies as a case of reference-point reasoning, whereby one prominent element is verbally used to refer and gain mental access to the envisaged target concept, which is more complex or harder to name and which unlike metaphor is situated within the same domain of experience. Some classic examples are presented in (4)–(5), where the *steak béarnaise* and the *crown* are verbal expressions that clearly refer to the costumer and the royal institution as a whole, but in both cases linguistically only profile a prominent (visible) part of it. Apart from this part-whole relationship as illustrated in (4)–(5), the category of metonymy comprises several other contiguity relationships like location for institution (6), whole for part (7), cause for effect (8), etc.

(4) The *steak béarnaise* wants to pay
(5) We swear loyalty to the *crown*
(6) *London* asks to postpone the brexit (referring to the British government)
(7) Where are *you* parked? (referring to the car)
(8) Have you fallen on your head? (referring to someone’s particular state of mind as being caused by the falling

The third semantic mechanism, polysemy, pertains to all kinds of related meanings connected by a single verbal form, which do not qualify as either metaphor or metonymy, as in (9), where two different but related meanings of *key* are realized. Next to these three semantic mechanisms metaphor, metonymy, and polysemy, we hypothesized that other semantic mechanisms like wordplay and meaning associations through intertextuality, which in a previous study was found to favour humorous reinterpretations in spontaneous interactions (Feyaerts 2013) would be identified as well as semantic mechanisms operating humorous cognitive reappraisals. By wordplay we generally mean the witty exploitation of aspects of form and/or meaning in all kinds of verbal expressions, as in the one-liner in (10), attributed to Tom Waits. Through its reference to another text, character, play, music piece, movie, etc., the phenomenon of intertextuality may also trigger a new (humorous) interpretation of a specific utterance, as in (11).

(9) The key broke in the lock vs. The key problem was not one of quality but of quantity
(10) ‘Champagne for my real friends and real pain for my sham friends’
(11) Then she realized this would be the right moment for another ‘I’ll be back!’ (intertextual reference to Arnold Schwarzenegger’s iconic character of Terminator)

In addition to these five semantic mechanisms and in line with findings of a previous study on spontaneous humor processing (Winchatz and Kozin 2008; Feyaerts 2013), we also expected three conceptual dimensions to be involved in an interpretational shift to a humorous meaning: different world of experience, fantasy and fiction, narrative change of perspective. Whereas the first two pertain to the creation of an incongruous interpreting experience as one of the preconditions, which are often required to generate a humorous meaning, the change of narrative perspective as apparent in switching from an objective description to a subjective first-person narrative, already by itself activates an additional layer of meaning. The utterance in (11), where the quote from Schwarzenegger’s Terminator introduces an unexpected, rather humorous interpretational shift, provides a nice illustration of this. We refer to “Extraction of Semantic and Conceptual Mechanisms” section for a comprehensive and contextualized description of the identified linguistic mechanisms along with a discussion of examples from the present corpus.

Fourthly, to further evaluate the effectiveness of these mechanisms in humor creation, their use was correlated with humor creation performance on the level of the individual. We expected that the use of metonymy in particular, which is a potent tool to build relationships between conceptual entities that are incongruous at first sight, may enhance the quality of humor creation (Tabacaru and Feyaerts 2016). The identification of these various relevant linguistic mechanisms provides a realistic, empirically substantiated account of the internal semantic relationships along whose lines humorous reappraisals can be construed. Together, in addition to novel basic-research evidence relevant to the fields of humor research, affective science, creativity research, and cognitive linguistics, the study aimed at providing empirical information for the (further) development of therapeutic interventions implicating positive re-interpretations or humor for better coping with stress and adversity.

## Methods

### Participants

The study sample comprised *n* = 101 participants (60 women, 41 men; age range 17 to 64 years, *M* = 23.5, *SD* = 9.3). Levels of education were: less than high school (33), high school graduate (45), university degree (23). None of the participants reported using psychoactive drugs or medication and none had participated in an experiment using the Reappraisal Inventiveness Test before (exclusion criteria). The study was advertised via social media and posters at several university and high school and college campuses. Interested individuals were contacted to check for exclusion criteria and arrange an appointment. Two interested individuals were not tested because they reported taking antidepressive medication (SSRI). Five interested individuals were not tested because they had participated in an experiment using the Reappraisal Inventiveness Test before. Five individuals who had an appointment failed to show up at the agreed time. The study was approved by the authorized ethics committee. Informed consent was obtained from all individual participants included in the study.^2^

### Humor Creation Task

For the purpose of studying humor creation in the context of cognitive reappraisal, the Reappraisal Inventiveness Test (RIT; Weber et al. 2014; see also Fink et al. 2017; Papousek et al. 2017; Perchtold et al. 2018; Rominger et al. 2018) was adapted to use it with a humor instruction (HRIT). Four vignettes of the Reappraisal Inventiveness Test depicting anxiety-eliciting situations (de Assuncao et al. 2015; Perchtold et al. 2019; Weber et al. 2014) were presented one at a time on separate pages along with a picture in order to make them more vivid. In the office item, for instance, participants faced the following situation: “Late at night, you are the only one left working at the office. As you are sitting at your desk, suddenly all the lights on your floor switch off”. In line with the standard instructions of the Reappraisal Inventiveness Test (Weber et al. 2014), participants were instructed to imagine the situation happening to them, and were given 20 s to immerse themselves in the situation and then turn to the next page at the signal of the experimenter. Then they wrote down their ideas until the allotted time of 3 min per situation had elapsed. Participants were instructed to write down as many different humorous ways as possible to think about the situation in a way that may diminish their anxiety in this situation.

### Quantification of Humor Creation Performance

#### Quantity (Fluency in Humor Creation)

To determine to which extent the participants succeeded in producing (explicable) humor as objectively as possible, each reappraisal idea was rated according to the agreement of three authors (C.R., K.F., I.P.) whether it followed an identifiable humor structure. 1295 ideas were rated in total. Ideas were rated as “humorous”, if they could be classified as either [a] incongruity-resolution humor, comprising an unexpected incongruity which could be resolved through the punch line (142); [b] nonsense humour, comprising an incongruity which was left unresolved (75); [c] disparagement humour without typical incongruity, including sarcastic, cynical, and scoffing statements (260) (see Ferguson and Ford 2008; McGhee et al. 1990; Hempelmann and Ruch 2005; Platt and Ruch 2014). They were rated as “non-humorous” reappraisals if they [d] had no identifiable humorous structure, i.e., comprised no humour in the classical sense (818). Importantly, this classification focused on the linguistic structure of the reappraisals and was conducted independently from the extent to which the authors perceived them funny or the subjective funniness ratings of other raters. Internal consistency across the four vignettes was α = .61. In total, 1217 ideas were valid reappraisals according to the standard criteria of the Reappraisal Inventiveness Test (Weber et al. 2014). Reappraisals were scored by two independent, trained co-workers who were not identical to the authors rating the humor structure and not identical to the raters of funniness (i.e., three independent groups of researchers rated the humor structure, funniness, and reappraisal nature of the ideas, respectively), α = .71, ICC = .97.

#### Quality (Funniness of Created Humor)

The funniness of ideas classified as humor (477 in total) was rated by eight independent raters (author C.M.P. and 7 trained co-workers) on a scale from 0 (not funny at all) to 3 (extremely funny). To enable as objective evaluations as possible, humor responses were transcribed to an electronic file and alphabetically sorted across all participants per vignette before they were rated. The interrater-reliability was ICC = .73. The high number of raters was chosen to compensate for the great inter-individual variability in the perceived funniness of specific humorous material. The ratings were averaged across raters and across all valid humor responses of an individual.

### Extraction of Semantic and Conceptual Mechanisms

In accordance with the overview and the derived hypotheses presented in the introduction, in this section we offer a comprehensive and contextualized description of the linguistic mechanisms along with pertinent examples, as they were identified in the reported study’s reappraisal ideas that qualified as humorous.

#### Metonymy

In the introductory section we already described metonymy as a contiguity-based conceptual mechanism of reference-point reasoning, which operates within the borders of a single conceptual domain (Langacker 1993; Radden and Kövecses 1999; Feyaerts 1999, 2003; Koch 1999; Peirsman and Geeraerts 2006; Tabacaru and Feyaerts 2016). In contrast to the mainstream cognitive linguistic view, we regard the often cited criterion of just one knowledge domain being involved as an epiphenomenon of the contiguity relation, which includes associative-functional relationships such as cause-effect, container-contained, producer-product, part-whole, substance-object, etc. (see examples (4)–(8) in the introduction). In this diversity, metonymy differs from metaphor, in which the structural mapping between domains basically reduces to a relation of (conceptually embedded) similarity (A is like B; Feyaerts 1999; Barnden 2010). As pointed out by Dirven (1993), among others, contiguity essentially qualifies as *conceptual* contiguity as it does not need to be based on any form of objective or natural contiguity. Instead, it may be realized “when we just ‘see’ contiguity between domains” (p. 14).

Especially with regard to metonymic structures in humorous contexts, this conceptual predicate is of central importance as humor often hinges on the interpretation of unexpected, unreal(istic) or, for that matter, ‘incongruous’ relationships between conceptual entities (Tabacaru and Feyaerts 2016). In the following examples (12) and (13), taken from the corpus of the present study, participants refer to the threatening situation of a dangerously cracking suspension bridge in terms of what quite unexpectedly may have caused it: the participant has eaten too much cake, or the bridge must have been built by the trainee at the engineering company. In both cases, an incongruous cause-effect relation is construed, thus triggering a humorous re-interpretation of the described scene. Similar, the response in example (14) profiles an unexpected, incongruous cause for the described situation of noticing that one is being followed on the streets at night. In this situation, in which threatening stalking behavior is suggested, the response induces a humorous re-interpretation of the entire scene in terms of a famous person being chased by a paparazzo.

Finally, the response in example (15) is yet another example of an expression that metonymically refers to the expected catastrophe of the suspension bridge collapsing. This time, however, the humorous meaning shift does not involve profiling the event’s unexpected cause as in (12) to (14) but one of its possible consequences. In this case, the verbally coded effect is an expression of relief over the fact that one is wearing fresh underwear, which as such refers to a widespread, somewhat stereotypical educational practice of reminding children to always wear fresh underwear in case one ends up being caught in some accident, as a consequence of which people at the hospital need to remove one’s clothes (and get to see one’s underwear ...).

(12) *Oje, das letzte Stück Kuchen war wohl zu viel!* [Oops, I guess that last piece of cake was too much].
(13) *Hat sicher der Praktikant gebaut.* [Probably built by a trainee].
(14) *Ein Paparazzo.* [A paparazzo].
(15) *Gut, dass ich frische Unterwäsche trage!* [So good that I am wearing fresh underwear!].

#### Metaphor

In line with the vast cognitive linguistic literature on conceptual metaphor theory, metaphor is defined as a systematic, similarity-based mapping of concepts across different domains of experience (see introduction). In the following example (16) participants are confronted with a threatening situation, in which suddenly during night work all the lights in the office building are switched off, except for the one at one’s own desk. Examples (17) and (18) refer to the scene in which one notices being followed on the streets at night. In taking an unexpected metaphorical perspective on (parts of) the scene, participants install some form of incongruity, which briefly de-automatizes the ongoing interpretation process and allows the activation of a humorous interpretation of that scene (Brône and Feyaerts 2003; Tabacaru and Feyaerts 2016). In example (16) the participant presents herself as an (electrical) machine of which due to some system overload the fuse powering the brain (machine) has been burnt. The humorous power in (17) hinges on the verbally implicit yet prominent conceptualization of the participant as a witch who is looking for her broom in order to fly away from the scene. Finally, in (18) the participant attempts to neutralize and motivate the threat by objectifying herself as the follower’s GPS system.

(16) *Mein Gehirn steht so unter Strom, dass eine Sicherung rausgeflogen ist.* [My brain gets so much power that a fuse was blown].
(17) *Wo ist eigentlich mein Besen, wenn ich ihn brauch’*. [Where is my broom when I really need it].
(18) *Ich bin wohl sein Navi.* [I guess I am his GPS].

#### Polysemy

Different meanings of a single word form may be related through semantic relations other than metaphor or metonymy, as is the case with generalization and specification. In this study, these other types of semantic relations were categorized as instances of polysemy. They are to be distinguished from cases of homonymy, which refers to two different words that only share a common word form without the meanings being related, as in bank ‘piece of furniture meant for sitting’ vs. bank ‘financial institution’. In the reappraisal formulated in example (19), in which the participant directly addresses the dangerously cracking wooden suspension bridge, the German verb ‘krachen’ refers both to the loud cracking sounds produced by the bridge and the figurative meaning of ‘partying’ as in ‘let’s rock’. Another example is (20), where the usage of the expression ‘lass dich nicht so hängen’ [‘hang in there’] in a situation of crossing a cracking wooden bridge allows the activation of the original literal meaning as well.

(19) *Nur los, lass es krachen.* [Let’s rock].
(20) *Komm schon, lass dich nicht so hängen, Brücke!* [Come on, bridge, hang in there].

#### Word Play

In line with Delabastita (1993), we categorize as word play a wide range of form similarity, including form identity, between at least two linguistic structures coupled with more or less dissimilar meanings. Attardo (1994) specified that the meanings involved in punning must be in opposition to one another and that the ambiguity must be deliberately pointed out. In the following examples (21) and (22) participants make use of word play as a mechanism to neutralize the threat of the cracking suspension bridge. In example (21), where the fixed expression ‘alles schwingt und singt’ [‘everything rocks and sings’], which is normally used to refer to a pleasant (musical) experience, is altered into a mildly cynical expression by using the German equivalent for ‘sinking’. Similar, the use of the English expression ‘hanging around’ in example (22) leads to a humorous effect in the context of a dangerously cracking suspension bridge. In example (23), the single-word question ‘Power nap?’ can be interpreted conventionally as a ‘short sleep meant for revitalization that is terminated before deep sleep’ or, as in the threatening situation of all lights in the office building suddenly going out, funny and unconventionally as ‘the (personalized) power taking a nap’.

(21) *Alles schwingt und sinkt.* [Everything rocks and sinks].
(22) *Just hanging around.*
(23) *Power nap?*

#### Intertextuality

In some cases, a rather unexpected and humorous interpretation of the threatening scene is obtained through the intertextual evocation of a character, prop, scene, or the music from a well-known video, movie, or play. In example (24) below, the quote of the first line of Carl Douglas’ famous hit Kung Fu Fighting (‘Everybody was kung fu fighting’) allows a sudden interpretational shift of the participant from a frightened designated crime victim into a powerful self-acting fighting machine. In example (25), which refers to the situation in which all lights in the office building suddenly went out, the mentioning of a magic wand with the accompanying spell that may be used to ‘bring maximal light’ projects the threat into the playful world of Harry Potter.

(24) *Everybody was kung fu fighting!*
(25) *Zauberstab → LUMOS MAXIMA*. [Magic wand *→* LUMOS MAXIMA].

#### Different World of Experience

Participants may situate the threatening situation against a totally different conceptual background, thus triggering an unexpected, potentially humorous re-interpretation. In example (26) the alleged stalker is assumed to be willing to dance, whereas in (27) all other except one’s own lights going out is re-interpreted in terms of a theatre’s spot light highlighting the main actor. Similar, in example (28) the cracking wooden floor of the suspension bridge is interpreted in terms of a beautiful rhythmic pattern, which nicely matches the sound of the tearing cables.

(26) *Er will wohl eine Polonaise starten*. [I guess he wants to start dancing a polonaise].
(27) *Schön, dass ich im Rampenlicht bin.* [Nice that I am in the spot lights].
(28) *Das Knirschen der Bretter passt rhythmisch perfekt zum Reißen der Seile.* [The cracking of the blanks perfectly fits the tearing cables].

#### Fantasy and Fiction

In case participants generated a reappraisal idea as in examples (29) to (32), in which the humorously re-interpreted threatening situation was projected into a world of fantasy and fiction, it was categorized accordingly. Example (29) refers to the scene in which suddenly during night work all lights are switched off, whereas examples (30) and (31) refer to the situation in which one notices being followed on the streets at night. Example (32) was created after confrontation with the scene of a dangerously cracking suspension bridge.

(29) *Zauberstab → LUMOS MAXIMA*. [Magic wand → LUMOS MAXIMA].
(30) *Wo ist eigentlich mein Besen, wenn ich ihn brauch’*. [Where is my broom when I really need it].
(31) *E.T., bist du es?* [E.T., is that you?]
(32) *Spider Man, wo bist du?* [Spider Man, where are you?]

#### Narrative Change of Perspective

Reappraisal ideas were coded as a narrative change of perspective in case participants construed a communicative situation in which they directly, as one of the protagonists, entered a dialogue. Accordingly, the examples (33) to (35) express a question, request, and a wish, respectively. The participant in example (35) refers to the expected crash of the suspension bridge.

(33) *E.T., bist du es?* [E.T., is that you?]
(34) *Spider Man, wo bist du?* [Spider Man, where are you?]
(35) *Na dann, guten Flug.* [Well then, have a nice flight].

### Individual Differences Variables

Depressive symptoms were assessed by the Center for Epidemiologic Studies Depression Scale (CES-D, German version; Hautzinger and Bailer 1993). It is comprised of 20 items, rated from 0 to 4 (Cronbach’s alpha α = .91). The CES-D is designed for measuring sub-clinical depressive daily-life experiences in the general population (Wood et al. 2010). Scores ranged from 1 to 48 (*M* = 15.7, *SD* = 10.0).

Seriousness as a trait was assessed by the State-Trait-Cheerfulness Inventory (STCI-T < 60>, German trait version, Ruch et al. 1996; see also Ruch and Hofmann 2012). The seriousness subscale of the STCI-T < 60> comprises 20 items rated from 1 to 4 (α = .81, *M* = 48.2, *SD* = 7.8; *min* = 28, *max* = 67). High scores indicate a serious frame of mind including an earnest, sober, non-playful attitude (Ruch and Köhler 2007; Ruch et al. 1996).

For the assessment of participants’ current negative mood a German short version (Dalbert 1992) of the Profile of Mood States (POMS; McNair et al. 1971) was used. Participants rated on scales from 1 to 7 how they felt at the moment (19 items, α = .92, *M* = 57.8, *SD* = 10.2; *min* = 35, *max* = 90; higher scores denote a more negative mood state).

After completion of all vignettes, they were presented once again, and participants rated for each of the four depicted situations the degree of anxiety they would experience when confronted with this situation (7-point scales ranging from 0 ‘not anxious’ to 6 ‘extremely anxious’). In one sample *t*-tests, ratings for all vignettes differed significantly and markedly from zero (*t*-values ranging from 15.9 to 21.6, all *p*-values < .001), which confirms that all situations were indeed perceived as anxiety evoking. The average rating was *M* = 3.0 (*SD* = 1.1; *min* = 0.25, *max* = 5.25; α = .54), with high scores indicating high perceptions of threat, indicating that in line with the conceptualization of the RIT, the situations were perceived as moderately stressful.

### Procedure

After receiving general instructions, participants completed the POMS, the adapted Reappraisal Inventiveness Test, the anxiety ratings, the CES-D, and the STCI-T. In a subsample of the present study sample, afterwards additional data were obtained for purposes not related to the present research questions. These included habitual comic styles (Ruch et al. 2018) and maladaptive personality traits (Zimmermann et al. 2014). Participants were tested singly or in small groups in a quiet, undisturbed room.

## Results

### Quantity of Humor Production

**Fig. 1:**
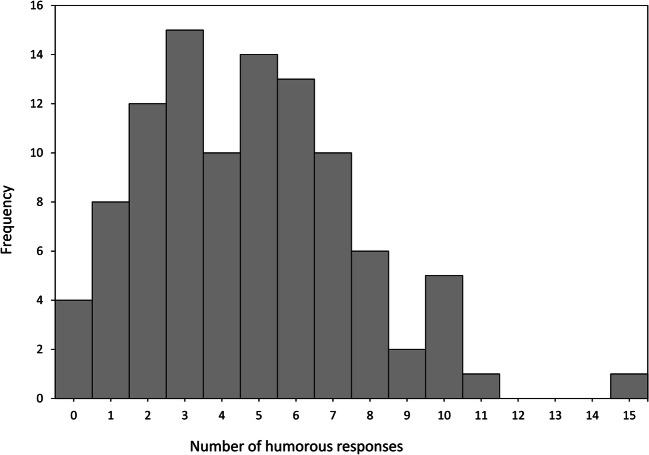
Quantity of humor production: Number of humorous ideas

On average, each participant produced *M* = 4.72 (*SD* = 2.83; 95% CI [4.17, 5.28]) humorous ideas in response to the four vignettes of the humor creation task (*min* = 0, *max* = 15). Only four of the total 101 participants (3.96%; 95% CI [0.16, 7.76]) did not manage to create any valid humorous responses. The distribution of the number of produced humorous ideas is shown in Fig. 1. Viewed on a relative basis, on average *M* = 37.92% (*SD* = 20.73; 95% CI [33.83, 42.01]) of all responses produced by a participant were classified as humorous according to the criteria explained in “Quantity (Fluency in Humor Creation)” section (*min* = 0%, *max* = 93.75%). *M* = 85.71% (*SD* = 20.72; 95% CI [81.54, 89.89]) of all humorous responses of a participant were at the same time valid cognitive reappraisals according to the standard criteria of the Reappraisal Inventiveness Test (Weber et al. 2014; *min* = 0%, *max* = 100%).

Correlations of a participant’s fluency in humor creation with depressive symptoms (*r* = −.11, *p* = .259), seriousness (*r* = −.07, *p* = .466), negative mood state (*r* = −.05, *p* = .615), and perception of the situations as more threatening (*r* = −.01, *p* = .953) were all non-significant (*n* = 101). The correlation between the quantity of humor production and the total number of generated ideas was *r* = .30 (*p* = .002). Men and women did not differ in the quantity of produced humor (*t*(99) = 0.7, *p* = .460).

### Quality of Humor Production

**Fig. 2:**
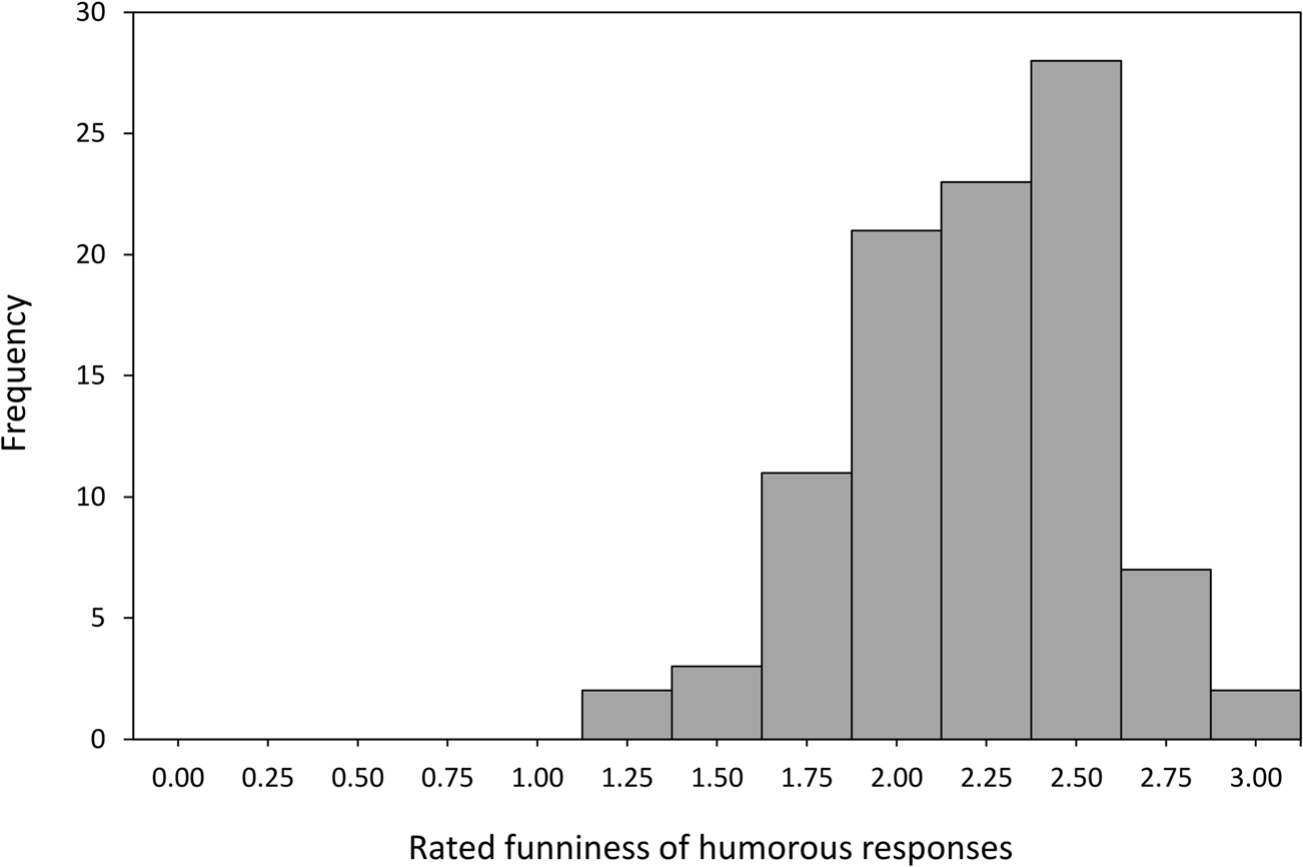
Quality of humor production: Average funniness of a participant’s humorous ideas

The average rated funniness of a participant’s responses classified as humor was *M* = 2.21 (*SD* = 0.36; 95% CI [2.14, 2.28]; *min* = 1.25, *max* = 2.95, on a scale from 0 to 3). The distribution of funniness ratings is depicted in Fig. 2 (participant basis, *n* = 97). The funniness of an individual’s created humor did not correlate with depressive symptoms (*r* = −.04, *p* = .683) and negative mood state (*r* = −.13, *p* = .210). Funniness ratings were higher in individuals scoring lower on seriousness (*r* = −.21, *p* = .043). Moreover, participants produced funnier responses when they had perceived the situations as more threatening (*r* = .28, *p* = .006). Related to that, there was a positive correlation of *r* = .19 (*p* = .057) between the anxiety rating and the percentage of humorous responses that at the same time were rated as valid reappraisals of the situation. The difference between men (*M* = 2.13, *SD* = 0.35) and women (*M* = 2.26, *SD* = 0.36) was non-significant (*t*(95) = 1.73, *p* = .088). The correlation between fluency of humor creation (number of humorous ideas) and quality (funniness) was *r* = .17 (*p* = .090).

### Semantic Mechanisms

**Fig. 3:**
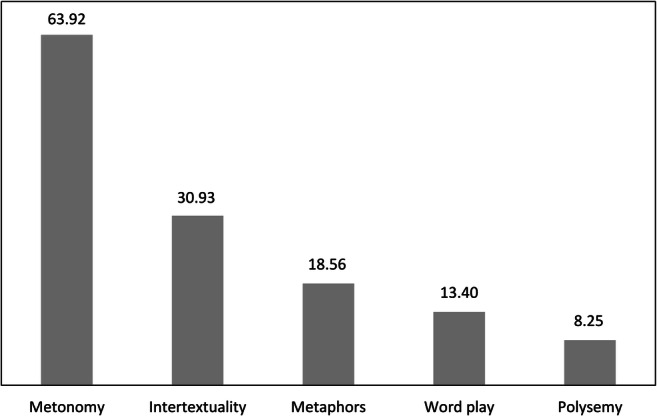
Percentage of participants using linguistic mechanisms

Of the 477 humorous responses that were produced in the sample in total, 117 contained metonymies, and 42 involved intertextuality. Metaphor (24), word play (15), and polysemy (9) occurred more rarely. On the level of the individual, of those who produced humor at all (*n* = 97), *n* = 62 participants (63.92%; 95% CI [54.36, 73.48]) used metonymy at least once. Intertextuality was used by *n* = 30 participants (30.93%; 95% CI [21.73, 40.13]). *N* = 18 (18.56%; 95% CI [10.82, 26.30]) of the participants used metaphor. Only relatively few participants used word play (*n* = 13, 13.40%; 95% CI [6.62, 20.18]), and polysemy (*n* = 8, 8.25%; 95% CI [2.78, 13.73]; Fig. 3).

Individuals drawing on the mechanism of metonymy when creating humor (at least in one of their ideas) produced more humorous ideas in total (*M* = 5.45, *SD* = 2.59) compared to individuals who did not use this mechanism (*M* = 3.97, *SD* = 2.70; *t*(95) = 2.7, *p* = .009), and their humor was on average rated funnier (*M* = 2.27, *SD* = 0.31; *M* = 2.09, *SD* = 0.40; *t*(95) = 2.45, *p* = .016). The same applies to the mechanism of intertextuality. Individuals drawing on this mechanism produced more humorous ideas in total (*M* = 6.43, *SD* = 2.61; *M* = 4.24, *SD* = 2.49; *t*(95) = 3.96, *p* < .001) and their humor was rated funnier (*M* = 2.38, *SD* = 0.22; *M* = 2.13, *SD* = 0.38; *t*(95) = 3.26, *p* = .002) compared to individuals who did not use intertextuality in their humor production. Metaphors, too, were employed more likely by participants who were more fluent in their humor production (*M* = 6.61, *SD* = 3.40; *M* = 4.53; *SD* = 2.39; *t*(95) = 3.06, *p* = .003) but their humor was not rated funnier compared to participants not using metaphors (*M* = 2.26, *SD* = 0.25; *M* = 2.20, *SD* = 0.38; *t*(95) = 0.69, *p* = .495). The same picture emerged for the two more rarely used linguistic mechanisms. Individuals drawing on word plays or polysemy were more fluent in producing humor in total but the quality of the humor did not differ from that of participants not using these mechanisms (word play: *M* = 7.15, *SD* = 2.27; *M* = 4.57, *SD* = 2.62; *t*(95) = 3.36, *p* = .001; *M* = 2.32, *SD* = 0.28; *M* = 2.19, *SD* = 0.37; *t*(95) = 1.26, *p* = .210; polysemy: *M* = 7.00, *SD* = 1.69; *M* = 4.73, *SD* = 2.71; *t*(95) = 2.32, *p* = .022; *M* = 2.31, *SD* = 0.45; *M* = 2.20, *SD* = 0.35; *t*(95) = 0.86, *p* = .391).

### Conceptual Phenomena

**Fig. 4:**
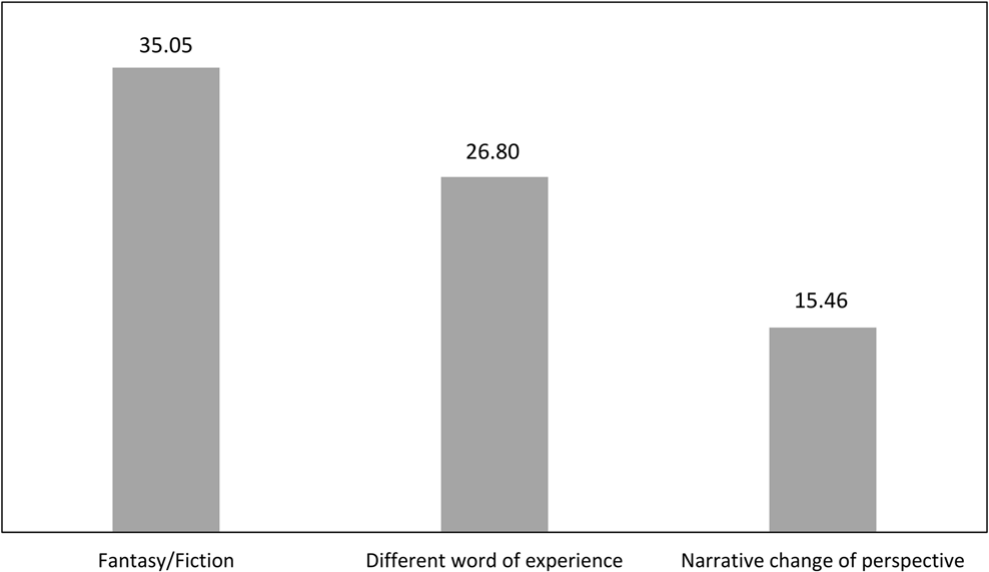
Percentage of participants employing conceptual phenomena

Fifty-three humorous responses drew on fantasy/fiction, 34 on different worlds of experience, and 19 on a narrative change of perspective. On the level of the individual, *n* = 34 (35.05%; 95% CI [25.56, 44.55]) used fantasy/fiction in at least one of their humorous ideas, *n* = 26 (26.80%; 95% CI [17.99, 35.61]) used different worlds of experience, and *n* = 15 (15.46%; 95% CI [8.26, 22.66]) used a narrative change of perspective (Fig. 4).

Individuals drawing on fantasy/fiction (*M* = 6.74, *SD* = 2.79) or narrative changes of perspective (*M* = 6.40, *SD* = 3.72) were more fluent in their overall humor production compared to participants who did not use these elements (*M* = 3.94, *SD* = 2.11; *t*(95) = 5.56, *p* < .001; *M* = 4.65, *SD* = 2.42; *t*(95) = 2.36, *p* = .020). The quantity of produced humor did not differ between individuals having used and not used a different world of experience in their humorous ideas (*M* = 5.27, *SD* = 2.39; *M* = 4.79, *SD* = 2.82; *t*(95) = 0.77, *p* = .442). Employment of the more content-related elements did not significantly affect the average quality (funniness) of an individual’s humor (fantasy/fiction: *M* = 2.23, *SD* = 0.37; *M* = 2.20, *SD* = 0.35; *t*(95) = 0.41, *p* = .681; different world of experience: *M* = 2.20, *SD* = 0.35; *M* = 2.21, *SD* = 0.36; *t*(95) = 0.2, *p* = .842; narrative change of perspective: *M* = 2.35, *SD* = 0.25; *M* = 2.18, *SD* = 0.37; *t*(95) = 1.68, *p* = .096).

## Discussion

The present study examined the ability to create humor in the context of efforts to positively re-interpret threatening situations. To obtain a valid indicator of the fluency (quantity) aspect of humor creation, responses were classified as successfully humorous if they followed a pre-defined, identifiable humor structure, uninfluenced from subjective perceptions of funniness, which was independently rated for gaining a quality indicator. Additional research questions addressed whether the basic capability to create humor in the context of cognitive reappraisal may be compromised by several potentially relevant traits and states of the individual, and the analysis of linguistic mechanisms that may be helpful in humor creation.

On average, participants were able to produce a remarkable number of spontaneous humorous ideas, and only four out of 101 participants did not come up with any humor. Moreover, almost all humorous responses were at the same time valid cognitive reappraisals according to the standard category system of the Reappraisal Inventiveness Test (Weber et al. 2014). Thus, mixing re-interpretations of stressful situations with humor does not seem to entail too much difficulty. Even more importantly, the quality of the created humor in terms of funniness was rated markedly higher than typical funniness ratings of responses in the most widely used cartoon-caption test for the assessment of humor production ability (Cartoon Punch Line Production Test; Kellner and Benedek 2017; Ruch and Köhler 2007; Ruch et al. 2009). This is most likely owed to participants’ higher motivation in the more relevant task in the present study.

Quantity and quality seem to be two largely distinct aspects of humor creation. In the present study, the intercorrelation between the two aspects was low and not statistically significant. Only a few previous studies have assessed both the quantity and the quality of produced humor. The correlations between the two variables ranged from zero (Turner 1980) to moderately positive (Jurcova 1998) to somewhat higher correlations of around *r* = .50 (Köhler and Ruch 1996; Moran et al. 2014; for discussion see Ruch and Heintz 2019). One possible explanation for the heterogeneous findings are differences in time restrictions among the tasks. Findings of Derks and Hervas (1988) suggested that later ideas were more funny, if a greater number of humorous ideas were produced, which may result in a positive correlation between the quantity and the quality aspect of humor creation. However, the cartoon-caption task in that study was employed without time restrictions, and participants were a priori instructed to produce either 2 or 10 captions for an item. If individual differences in fluency are in the focus of interest, time limits are essential for their reliable measurement, even if they might to some extent affect the average quality of the responses in terms of their funniness or originality (see, e.g., Cheng et al. 2016; Rominger et al. 2018). Obviously, the 3-min time limit employed in the HRIT is short enough to prevent a confounding effect of quantity and quality.

From a linguistic perspective, the richness of the humorous re-interpretations in this study, brought about by the activation of different semantic mechanisms, is highly supportive of the cognitive linguistic view that meaning cannot be confined to a mere linguistic, system-inherent, and rigid value but rather qualifies as a usage-based, conceptually rich, and therefore dynamic structure. Along the lines of regular principles of meaning extension, individuals are capable of turning a threatening interpretation of a specific situation into a pleasurable humorous one, thus showing the pivotal role of the situationally and contextually embedded individual in the processing of meaning construal (see, e.g., Veale et al. 2006; Brône 2008 on the process of hyperunderstanding).

Metonymy emerged as by far the most prominent semantic mechanism in the creation of humorous re-interpretations, and its use was also related to good humor creation performance in terms of quality. This is in line with its assumed importance in the extension of meaning in general and the creation of humor in particular, and provides strong empirical support for claims made in earlier studies: In their corpus-based description of the rich variation patterns in two different clusters of expressive utterances – German insults for stupidity and ambiguous newspaper headlines – Feyaerts and Brône (2005) identified metonymy “as a cognitive mechanism of construal with a clear impact on non-referential (stylistic, emotive) aspects of meaning appreciation” (p. 29). In line with the difference in frequency between the activation of metaphor and metonymy in the creation of humorous re-interpretations in the present study, Feyaerts and Brône (2005) also observed that far more expressive and novel variants of German stupidity expressions were construed through metonymic rather than metaphorical extensions. The observation of metonymy as the most prominent semantic mechanism in humor creation is also in line with a corpus study of scripted interactional humor sequences. Tabacaru and Feyaerts (2016) identified metonymy and its conceptual chaining potential as an “excellent mechanism for the construction of stimuli that are innovative, expressive, or even plainly humorous, but which at the same time link back to a (partly) conventionalized meaning” (pp. 6–7). The almost absence of polysemy in the present study may be partially explained by the dominant role of metonymy and metaphor taken together as the most successful mechanisms of meaning extension. The observation that only few humorous ideas were construed along the lines of word play may be due to the fact that the task items consisted of descriptions and imaginations of threatening situations, without any focus on particular word forms. This, however, probably applies to most real-world situations (as opposed to perhaps more artificial test items).

Depressive symptoms or not being ‘in the right mood’ (i.e., a bad mood state) did not hamper humor creation in the context of cognitive reappraisal of stressful situations. In patients with depression, a lower self-rated tendency to use humor as a coping strategy in their daily lives was reported compared to healthy individuals (Falkenberg et al. 2011b). Anhedonia may result in reduced motivation to use humor in everyday life, similarly to the lack of motivation to display other behavior associated with otherwise positive or rewarding experiences (Dunn 2012). By contrast, the present study suggests that the basic ability to generate humorous ideas in the context of coping with adverse situations is unimpaired, at least with low to moderate levels of depressive symptoms. Previous studies had reported non-significant correlations between subclinical depressive symptoms and quality of humor creation as well as no difference in humor creation between adolescent patients with depression and controls in cartoon-caption tasks (Edwards and Martin 2010; Freiheit et al. 1998). Another study suggested that patients with major depression in remission were principally able to produce humor in the context of short-term emotion regulation attempts when confronted with sad pictures (Braniecka et al. 2019). Using an ability test in the psychometric sense, the present findings add to this literature by demonstrating that the basic potential of people with moderate depressive symptoms to use humor is intact in the context of efforts to find positive re-interpretations of stressful situations. Assumed difficulties to generate positive imagery (Grol et al. 2017) do also not seem to have much impact on the quality of humor produced in that particular context.

By contrast, seriousness, which is characterized by a conscientious, sober, non-playful attitude (Ruch et al. 1996) seems to affect humor creation to some extent. Participants who were more serious-minded produced ideas that were rated as less funny, but their basic ability to create humor (quantity of ideas that followed an identifiable humor structure) was unaffected. Previous research reported negative correlations of seriousness with the quality and quantity of humor creation in a cartoon-caption test (Ruch and Köhler 2007). The crucial difference here may be that humor production in the context of coping with stressful situations serves a reasonable purpose, which better suits more serious-minded individuals than the relatively purposeless creation of cartoon captions. The negative relationship of seriousness with the rated funniness of produced humor is in line with similar findings in the field of creativity, where it was shown that more positive attitudes toward creativity were related to the tendency to use more remote associations in the ideas. These in turn are perceived as more original by others, and more funny in the case of humorous ideas (Acar and Runco 2014; Tschacher et al. 2015).

The developers of the Reappraisal Inventiveness Test took great care that the levels of anxious arousal elicited by the vignettes were not too high; in order to make sure that the generation of cognitive reappraisals remains a meaningful endeavor (Sheppes et al. 2014; Weber et al. 2014). Indeed, the average ratings of how threatening the situations were perceived were exactly in the moderate range for which the test items were designed. In line with these intentions, individuals’ threat ratings did not correlate with the number of humorous ideas they created in order to positively re-interpret the situations. This replicates previous findings with the standard anger version of the test, which also yielded a zero correlation between the retrospectively rated intensity of anger elicited by the situations and the quantity of produced cognitive reappraisals (Weber et al. 2014). That is, neither the ability to generate positive re-interpretations nor the ability to generate humor in this particular context seems to depend on the level of emotional arousal, as long as it remains in the mild to moderate range. Note that most stressful situations in the daily lives of most people in the studied population can be characterized as mildly to moderately stressful.

While the perception of the situations as more stressful did not impede the quantity of humor creation, it even seemed to increase its quality. Participants who rated the situations as more anxiety-eliciting produced ideas that were funnier. The link to higher quality of ideas was further corroborated by the finding of a positive correlation between the intensity rating and the percentage of humorous responses that at the same time qualified as valid reappraisals of the situation. These findings are nicely in line with a study in which participants were required to generate a set of options for action in everyday life scenarios, a task which bears resemblance to the Reappraisal Inventiveness Test. Participants produced ideas that were more original and more divergent to familiar situations that they found more unpleasant (Schweizer et al. 2016). The perception of the situations as more stressful may facilitate the construction of larger incongruities, which are then experienced as more creative and funny by others (cf. Acar and Runco 2014; Tschacher et al. 2015). Through the related more pronounced switch of perspective, the responses perhaps also more likely qualified as cognitive reappraisal. However, further research is needed to clarify the mechanisms underlying this relationship between a more stressful nature of situations and higher quality of humor in ideas for their positive re-interpretation.

The options to analyze details of the structure and content of the created humor are one of several unique features of the specific test applied in the present study. An important distinctive feature of the HRIT is the classification of the responses based on whether they follow an identifiable humor structure at all. Typically, all responses are counted in humor production tasks, regardless of whether participants actually succeeded in creating humor. The fluency component as it was obtained in the present study may be more distinct from general verbal fluency and, hence, a more meaningful indicator of the quantity of humor creation. Note that the correlation between the quantity of humor creation and the total number of generated ideas was low. Only 9 % of the variance in the quantity of actual humor production were directly explained by the total fluency in generating (any) responses. A further important feature of the HRIT is that it is relevant and ecologically valid as it closely resembles a real-life situation in which one deliberately strives to cognitively reappraise a current stressful situation for effective coping. The most widely used humor production performance tests require participants to create funny captions for captionless cartoons (see Ruch and Heintz 2019 for review). While this is a potent task for assessing individual differences in humor production abilities, it is certainly far from what people are doing in their daily lives. One performance task assessed humor creation in the broader context of coping with stressful situations, though not specifically through immediate cognitive reappraisal (Edwards and Martin 2010). Participants were presented with descriptions of frustrating situations and were instructed to think of funny ways to later recount the experiences to a friend. The task allowed to analyze the quality but not the fluency of humor production. Previous investigations specifically addressing the use of humor in cognitive reappraisal focused on the potential positive effect of humor in this context, but their designs did not allow to study to which extent participants were actually able to produce humorous ideas (Kugler and Kuhbandner 2015; Samson and Gross 2012; Samson et al. 2014). A potential limitation is the relatively low number of items. While the psychometric properties of the RIT and HRIT scores typically are satisfying (see also Perchtold et al. 2019; Weber et al. 2014), it cannot be excluded that the low number of items may to some extent have induced underestimated sizes of relationships in the current study.

When it comes to more practical considerations, it is important to distinguish between humor creation in terms of how skilled or competent a person is at producing humor (most appropriately assessed by maximum performance tests; Edwards and Martin 2010; Ruch and Heintz 2019) and the production of humor as a typical or habitual behavior pattern (typically assessed by self-report scales). While humor creation as an ability captures what individuals are theoretically capable of, the production of humor as a habitual behavior captures how frequently the individual is inclined to use humor in everyday life. The fundamental ability to create humor in the context of the generation of cognitive reappraisals can be seen as a necessary but insufficient prerequisite for the effective implementation of humor in one’s everyday life coping with adverse circumstances. The same applies to cognitive reappraisal in general (Papousek et al. 2017; Weber et al. 2014), where it was also shown that individual’s basic capacity to generate cognitive reappraisals of stressful situations is uncorrelated with the typical frequency with which cognitive reappraisal is used as a coping strategy in daily life (Weber et al. 2014). For the effective use of humor in coping clearly both is required: the fundamental ability to create humor in this context as well as its implementation in daily life. Therapeutic interventions aimed at supporting clients in their use of humorous cognitive reappraisal may require that clients first develop their basic humor creation ability. Then, extensive practice in realistic situations will be important. Finally, with frequent repetitions in the same context, humorous appraisals may eventually become a habitual response to situations involving stress (Hertel 2004).

The study did not yield indications of major obstacles to the use of humor in the context of coping with stressful events in terms of individual characteristics or situational factors. While depressed persos may not be very much inclined to use humor (Falkenberg et al. 2011b), in the light of their apparently intact abilities, it may be possible to motivate depressed individuals to incorporate humor in their everyday coping efforts. The basic understanding of humor, evident from the detection of punch-lines and the cognitive comprehension of humor, also seems to be unaffected in depression, even if depressed patients do not seem to enjoy humor as much as healthy individuals (Falkenberg et al. 2011b; Horner et al. 2014). Impaired cognitive flexibility (Shilyasky et al. 2016) may perhaps be an obstacle to humor creation in patients with major depression. Yet, preliminary evidence suggested that the use of humor in coping can be increased by a targeted intervention and practice even in patients with major depression (Falkenberg et al. 2011a).

Individuals with a serious-minded attitude, too, seem to have an unimpaired ability to create humor in principle. However, similarly to depressed persons, they may need additional motivation for its implementation. With serious-minded individuals, it may be helpful to communicate that the intervention is not something they are doing ‘for fun’ but that it has a serious background and serves a reasonable purpose. Under these premises, people with serious-minded attitudes may be successfully instructed to use humor in their cognitive reappraisal efforts, with the caveat that their humor may be of lower quality and, hence, perhaps less effective in terms of changes of perspective and positive re-interpretation. However, the degree to which other people rate somebody’s ideas as funny is an insufficient indicator of how well the solution works for the person him- or herself. Further research is needed to determine whether individuals high on seriousness may benefit from the generation of humor in the context of coping to a similar extent as less serious-minded persons.

The identified linguistic mechanisms are examples for linguistic principles of how humorous reappraisals can effectively be constructed. Thus, they may be used as learnable tools in the context of humorous cognitive reappraisal of stressful situations. Drawing on fantasy or fiction and using a narrative change of perspective may additionally facilitate the creation of humor. The responses of individuals drawing on metonymy or intertextuality also had higher quality in terms of funniness. Hence, these linguistic strategies may be particularly valuable when it comes to effective cognitive reappraisal. The findings of the linguistic analysis may be used to create a pool of examples of humorous re-interpretations of stressful situations that may help therapists to support clients in learning and practicing the generation of humorous reappraisals.

To conclude, encouraging patients to use cognitive reappraisal constitutes the core of modern psychotherapeutic approaches. Hence, the findings of this study may provide useful empirical information for the (further) development of standardized therapeutic interventions aiming at increasing patients’ use of cognitive re-interpretations (e.g., Shore et al. 2017; Woud et al. 2012), which may benefit from the incorporation of humorous ideas. Likewise, they may provide useful information for the further development of interventions specifically targeting patients’ ability to use humor to better cope with stress and adversity (e.g., Falkenberg et al. 2013; Tagalidou et al. 2018). The HRIT, with simplified scoring, may be a suitable instrument for evaluating the success of such interventions, although its usefulness for this purpose has yet to be validated.

## Data Availability

The dataset generated during and analysed during the current study is available from the corresponding author on reasonable request.

## Footnotes

1 We side with cognitive linguistics, according to which linguistic meaning is essentially conceptually rich in nature involving both ‘proper’ linguistic, semantic relations and elements pertaining to the contextual and situational aspects of the usage-event, in which a linguistic element (a word, phrase, utterance, text, etc.) has been used. Accordingly, we regard the differentiation between semantic and conceptual relations not to be a categorical but rather an aspectual one.

2 Data from an overlapping but not identical sample, with an overlapping set of variables and different, non-overlapping research questions were published in Perchtold et al. (2019).
